# Ets-1 Is Essential for Connective Tissue Growth Factor (CTGF/CCN2) Induction by TGF-β1 in Osteoblasts

**DOI:** 10.1371/journal.pone.0035258

**Published:** 2012-04-23

**Authors:** Max T. Geisinger, Randy Astaiza, Tiffany Butler, Steven N. Popoff, Sonia Lobo Planey, John A. Arnott

**Affiliations:** 1 Basic Sciences Department, The Commonwealth Medical College, Scranton, Pennsylvania, United States of America; 2 Department of Anatomy and Cell Biology, Temple University School of Medicine, Philadelphia, Pennsylvania, United States of America; Northwestern University, United States of America

## Abstract

**Background:**

Ets-1 controls osteoblast differentiation and bone development; however, its downstream mechanism of action in osteoblasts remains largely undetermined. CCN2 acts as an anabolic growth factor to regulate osteoblast differentiation and function. CCN2 is induced by TGF-β1 and acts as a mediator of TGF-β1 induced matrix production in osteoblasts; however, the molecular mechanisms that control CCN2 induction are poorly understood. In this study, we investigated the role of Ets-1 for CCN2 induction by TGF-β1 in primary osteoblasts.

**Results:**

We demonstrated that Ets-1 is expressed and induced by TGF-β1 treatment in osteoblasts, and that Ets-1 over-expression induces CCN2 protein expression and promoter activity at a level similar to TGF-β1 treatment alone. Additionally, we found that simultaneous Ets-1 over-expression and TGF-β1 treatment synergize to enhance CCN2 induction, and that CCN2 induction by TGF-β1 treatment was impaired using Ets-1 siRNA, demonstrating the requirement of Ets-1 for CCN2 induction by TGF-β1. Site-directed mutagenesis of eight putative Ets-1 motifs (EBE) in the CCN2 promoter demonstrated that specific EBE sites are required for CCN2 induction, and that mutation of EBE sites in closer proximity to TRE or SBE (two sites previously shown to regulate CCN2 induction by TGF-β1) had a greater effect on CCN2 induction, suggesting potential synergetic interaction among these sites for CCN2 induction. In addition, mutation of EBE sites prevented protein complex binding, and this protein complex formation was also inhibited by addition of Ets-1 antibody or Smad 3 antibody, demonstrating that protein binding to EBE motifs as a result of TGF-β1 treatment require synergy between Ets-1 and Smad 3.

**Conclusions:**

This study demonstrates that Ets-1 is an essential downstream signaling component for CCN2 induction by TGF-β1 in osteoblasts, and that specific EBE sites in the CCN2 promoter are required for CCN2 promoter transactivation in osteoblasts.

## Introduction

Osteoblast growth, differentiation, and biosynthetic activity are initiated and tightly regulated by systemic and locally produced growth factors. Recently, connective tissue growth factor (CCN2), a 38 kDa, cysteine rich, extracellular matrix (ECM) protein that belongs to the CCN family of proteins, has emerged as an important growth factor in the control and regulation of osteogenesis [1] [2], [3], [4], [5]. CCN2 null (−/−) mice exhibit multiple skeletal dysmorphisms as a result of impaired growth plate chondrogenesis, angiogenesis, and bone formation/mineralization [6], and also exhibit numerous defects in the craniofacial, axial, and appendicular skeleton [7]. CCN2 is highly expressed in active osteoblasts lining osteogenic surfaces and is produced and secreted by osteoblasts in culture [2], [8]. CCN2 promotes proliferation, matrix production, and differentiation in osteoblasts [2], [5], [9], [10], [11], [12], [13], and CCN2 levels are stimulated by transforming growth factor-β1 (TGF-β1) [8], [13], [14], a finding that is consistent with a role for CCN2 in the effects of these proteins on skeletal growth [15]. TGF-β1 is a potent, multifunctional, osteogenic growth factor that also regulates osteoblast differentiation and function [16]. One of the major effects of TGF-β1 on osteoblasts is its ability to stimulate the production and secretion of ECM [17], [18], [19], [20], however the mechanisms or downstream effector genes that mediate this response are not understood. In osteoblasts, we recently demonstrated that CCN2 is stimulated by TGF-β1, and that CCN2 is a downstream effector for TGF-β1 induced ECM synthesis [8], [13], [14]. The signaling pathways that mediate TGF-β1 induction of CCN2 vary depending on the cell type being examined [21], and in osteoblasts they have only begun to be characterized. We have recently demonstrated that CCN2 protein induction by TGF-β1 in osteoblasts requires contributions of both the Smad and Erk signaling pathways [22], [23].

In general, TGF-β1 signals through a generic Smad mediated pathway involving Smads 2, 3, and 4 [24]. Smads 2 and 3 are phosphorylated by active transmembrane serine/threonine TGF-β1 receptors [25]. Following activation, Smad 2 and 3 form a trimeric complex with Smad 4, and this complex subsequently translocates to the nucleus, where it binds to Smad binding elements (SBE) in promoters of TGF-β1-responsive genes [24], [26]. Transcriptional activation by Smads is not limited to the Smad-SBE interaction alone but requires additional association of Smads with other transcription factors and co-factors that together bind the SBE and adjacent cis-regulatory binding elements (DNA motifs) [27]. We have previously demonstrated that in osteoblasts, the TGFβ response element (TRE/aka the BCE) in addition to the SBE, is essential for CCN2 promoter activation by TGF-β1 [22], [23]. However, the requisite additional transcription factors, co-factors, and DNA motifs required for Smad transcriptional activation are highly cell type dependent, and studies aimed at identifying these factors/motifs in osteoblasts are in their infancy.

We recently demonstrated that the MAPK, Erk, is also required for CCN2 induction by TGF-β1 in osteoblasts [22]. The requirement of Smad and Erk signaling to achieve CCN2 induction has also been demonstrated in other cells types [28], [29], [30], [31], [32], [33], [34]. Erk is known to potentiate the TGF-β1/Smad pathway via direct phosphorylation of Smads or indirectly through activation/inactivation of co-activators/co-repressors that mediate Smad DNA binding [35], [36]. We recently demonstrated that activation (phosphorylation) of Smads is not dependent on Erk, but that Erk phosphorylation is required for transcriptional complex formation on the SBE [23]. These results suggest that Erk mediates Smad signaling through activation of nuclear transcription factors that enhance Smad DNA binding. Activated Erk can translocate to the nucleus where it activates downstream transcription factors [37]. Erythroblastosis virus E26 oncogene homologue 1 (Ets-1) is a transcription factor that has been shown to be directly phosphorylated by Erk, and this leads to its activation and subsequent binding to Ets-1 binding motifs (EBE) in the promoters of target genes [37], [38], [39], [40].

Ets-1 is the founding member of the Ets family of transcription factors that control a wide variety of important biological processes including cell proliferation, differentiation, and ECM regulation [41]. Ets-1 is a downstream signaling effector for several TGF-β1 responsive genes [42], [43], [44], and more recently, Ets-1 has been shown to function downstream of TGF-β1 for CCN2 induction in fibroblasts [45]. Transcriptional activation by Ets-1 proteins functions in a combinatorial manner through association with other transcription co-factors to transactivate target genes [46], [47]. Recently, Ets-1 was found to synergize with Smad 3 to activate CCN2 expression in fibroblasts [48], and in separate studies, it was shown that Smad3 and Ets-1 co-immunoprecipitate and can act to form transcriptionally active complexes with the Smad transcriptional co-activators p300 and CBP [49], [50]. Ets-1 regulates transcription of target genes in a cell specific manner, and the role of Ets-1 for CCN2 regulation by TGF-β1 in osteoblasts is unknown. In this study, we investigated the contribution of Ets-1 for induction of CCN2 by TGF-β1 in osteoblasts.

## Results

### TGF-β1 induction of CCN2 requires Ets-1 in osteoblasts

**Figure 1:**
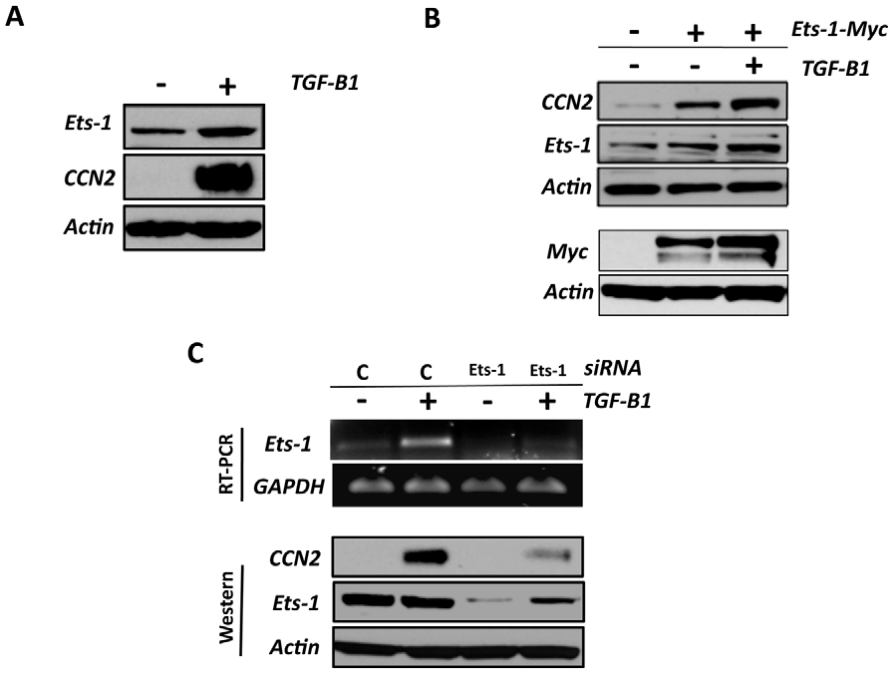
Ets-1 is essential for TGF-β1 induction of CCN2 protein in osteoblasts. (**A**) Primary osteoblasts were cultured until they were 80% confluent, serum deprived for 24 hrs and then treated with TGF-β1 (5 ng/ml) (+) or mock treated with TGF-β1 diluent (−). At 24 hrs post treatment, cell lysates were harvested and assessed for CTGF and Ets-1 expression by Western blot analysis. (**B**) Primary osteoblasts were transfected with Ets-1-Myc (+) or an empty vector (−) and then treated with TGF-β1 (5 ng/ml) (+) or mock treated with TGF-β1 diluent (−). At 24 hrs post treatment, cell lysates were harvested and assessed for CCN2, Ets-1 and Myc expression by Western Blot analysis. (**C**) Primary osteoblasts were transfected with 100 nM of Ets-1 siRNA (Ets-1) or control siRNA (C) for 48 hrs. Following transfection, the cells were serum starved for 24 hrs and then treated with 5 ng/ml of TGF-β1 (+) or TGF-β1 diluent (−) for 24 hrs. RNA was harvested and assessed for Ets-1 expression by RT-PCR. Cell lysates were harvested and assessed for Ets-1 and CCN2 expression by Western Blot analysis. Each experiment is representative of at least three independent experiments.

Ets-1 is a downstream signaling effector for several TGF-β1 responsive genes [42], [43], [44], and more recently Ets-1 has been shown to function downstream of TGF-β1 for CCN2 induction in fibroblasts [45]. In this study, we examined whether Ets-1 expression could be induced by TGF-β1 treatment of osteoblasts. The TGF-β1 dose of 5 ng/ml was used for this and all subsequent experiments, since we had previously demonstrated that this was the minimal dose required for maximal induction of CTGF in primary osteoblasts [13]. Using Western Blot and RT-PCR analysis, we found that Ets-1 protein levels (Figure 1A) and mRNA levels (see Figure 1C) were induced by TGF-β1 treatment of primary osteoblasts compared to treatment with TGF-β1 diluent alone, and that this was concomitant with CCN2 induction (Figure 1A). To determine if Ets-1 is required for CCN2 induction by TGF-β1, two approaches were used. For the first approach, we over-expressed Myc-tagged Ets-1 (Ets-1-Myc) and assessed CCN2 expression with or without TGF-β1 treatment (Figure 1B–C). For these experiments, primary osteoblasts were transiently transfected with either Ets-1-Myc or an empty (non-Ets-1 containing) vector. Ets-1over-expression was confirmed by Western Blotting using an anti-Myc antibody (Figure 1B). When we assessed CCN induction, we found that expression of Ets-1 induced CCN2 expression (Figure 1B), and that this expression was enhanced with both Ets-1 expression and TGF-β1 treatment. For the second approach, we used Ets-1 siRNA to impair Ets-1 expression at the protein and RNA level (Figure 1C). When we assessed CCN2 induction by TGF-β1 treatment in cells where Ets-1 was impaired, we found a significant reduction in CCN2 expression by TGF-β1 (Figure 1C), demonstrating the requirement of Ets-1 for CCN2 induction by TGF- β1 in osteoblasts.

### CCN2 promoter activation by TGF-β1 requires Ets-1

**Figure 2:**
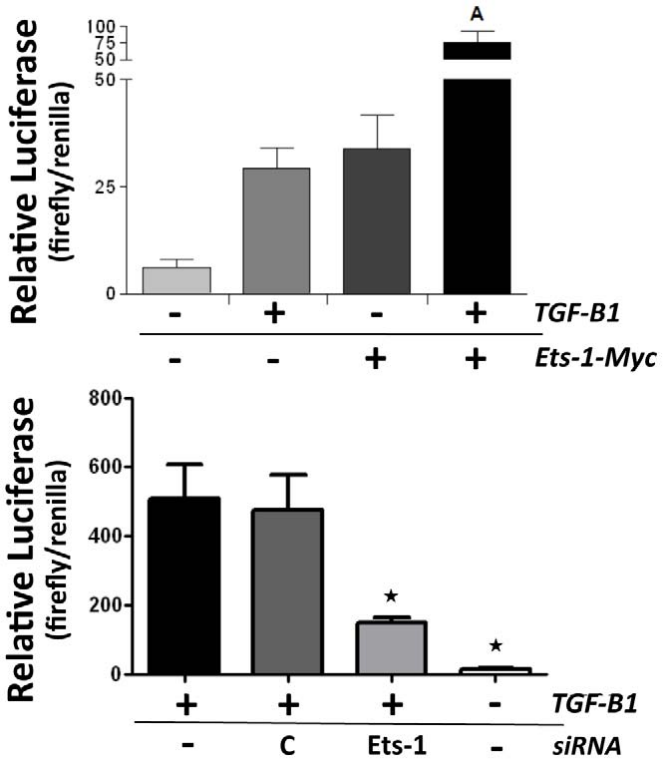
Ets-1 synergizes with TGF-β1 for CCN2 promoter induction in osteoblasts. (**A**) Osteoblasts were plated in 96 well tissue culture plates and transfected with either 0.4 µg of an empty vector control (−) or the Ets-1 expression construct (+). All samples were co-transfected with 0.4 µg of our previously described CCN2 promoter luciferase reporter [22] and 0.2 µg of a renilla luciferase expression vector as an internal control. The cells were serum starved for 24 hrs and then treated with TGF-β1 (5 ng/ml) (+) or mock treated (−) with TGF-β1 diluent for 24 hrs. Luciferase activity was then assessed and expressed as a ratio of firefly/renilla luciferase (+SEM, n = 6). A = p<0.05 compared to +TGF-β1 only or +Ets-1 only. (**B**) Osteoblasts were plated in 96 well tissue culture plates and transfected with either 100 nM of Ets-1 siRNA (Ets-1) or control siRNA (C) for 48 hrs. All samples were co-transfected with 0.4 µg of our previously described CCN2 promoter luciferase reporter [22] and 0.2 µg of a renilla luciferase expression vector as an internal control. The cells were serum starved for 24 hrs and then treated with 5 ng/ml of TGF-β1 (+) or mock treated (−) with TGF-β1 diluent for 24 hrs. Luciferase activity was then assessed and expressed as a ratio of firefly/renilla luciferase (+SEM, n = 6). Star symbol indicates p<0.05 compared to control siRNA.

In addition to CCN2 expression, we also assessed the requirement of Ets-1 for CCN2 promoter activation by TGF-β1 in osteoblasts. For these studies, we transfected osteoblasts with our previously characterized CCN2 promoter luciferase reporter [22] and either over-expressed Ets-1 (Ets-1-Myc) or blocked Ets-1 using Ets-1 specific siRNA (Figure 2). In Figure 2A, we found that Ets-1 over-expression alone induced CCN2 promoter activity at a level similar to TGF-β1 treatment, and that this promoter activity is significantly enhanced by simultaneous Ets-1 over-expression and TGF-β1 treatment (Figure 2A), suggesting that Ets-1 and TGF-β1 synergistically activate the CCN2 promoter. When we blocked Ets-1 with specific siRNA, we significantly impaired TGF-β1-induced CCN2 promoter activation (Figure 2B). These results demonstrate that Ets-1 is required for CCN2 protein induction and promoter activation by TGF-β1, and that Ets-1 synergizes with TGF-β1 to induce CCN2 in osteoblasts.

### Ets-1 binding elements (EBE) mediate TGF-β1 induction of the CCN2 promoter

**Figure 3:**
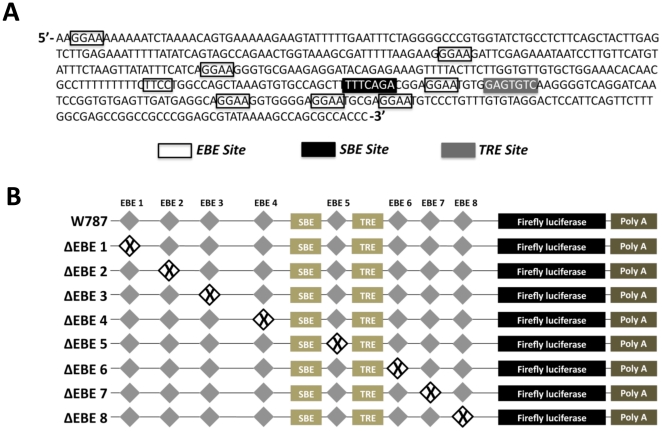
Identification of putative Ets-1 sites in the CCN2 promoter. (**A**) The CCN2 proximal promoter (pictured are bases −293 to +1) contains eight putative Ets-1 binding sites (EBE 1–8) (open boxes), and these sites are in close proximity to the TRE (grey box) and SBE (black box). (**B**) Multiple mutation constructs were created containing individual substitutions of the EBE at positions 1–8 (ΔEBE 1–8) in the CCN2 proximal promoter that was cloned into a pGL3-firefly luciferase reporter construct.

**Figure 4:**
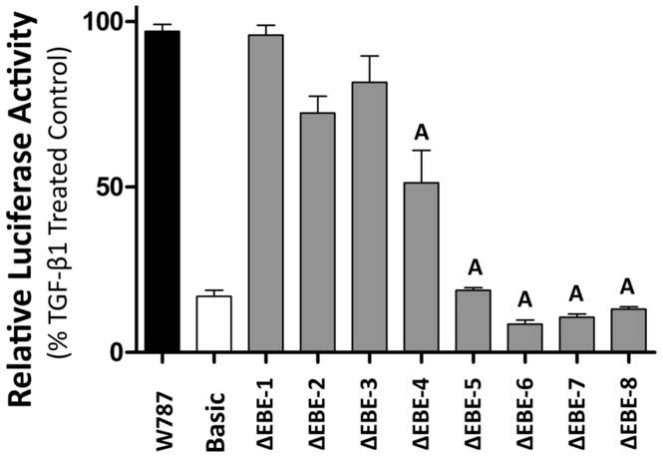
EBE sites are required for CCN2 promoter activation by TGF-β1 in osteoblasts. Osteoblasts were plated in 96-well tissue culture plates and transfected with 0.4 µg of either EBE mutation construct 1–8, pGL3-Basic (negative control) or W787 (positive control) and all were co-transfected with 0.2 µg of a renilla luciferase expression vector (internal control) for 24 hrs. The cells were serum starved for 24 hrs and then treated with TGF-β1 (5 ng/ml) for 24 hrs. Luciferase activity was assessed, and expressed as a % of activity obtained using the full length W787 construct. (+SEM, n = 6). A = p<0.05 compared to W787.

**Figure 5:**
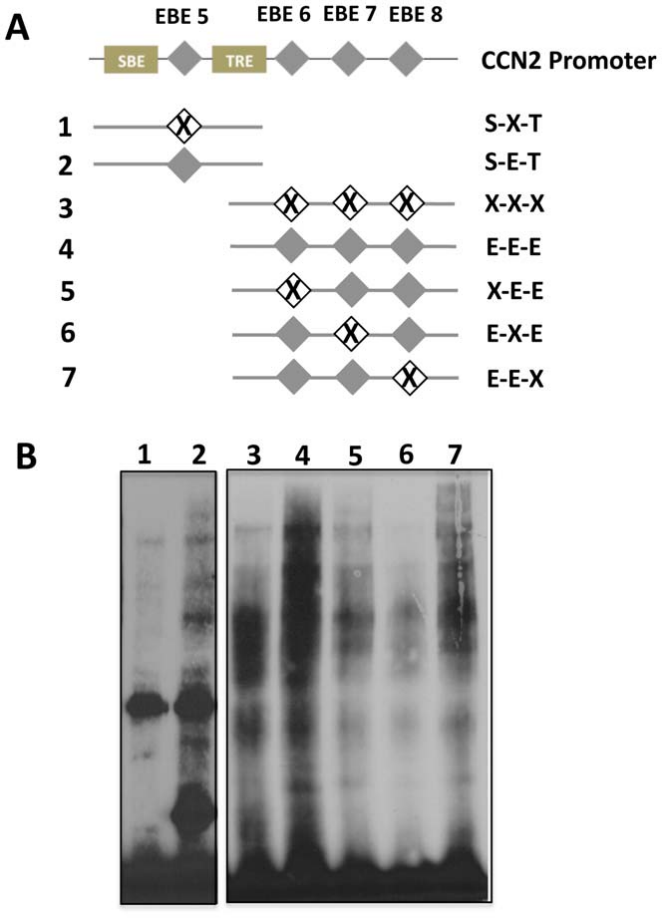
Mutation of EBE sites prevents protein complex binding. (**A**) Electro-mobility shift assays (EMSA) probes were created that were homologues to the CCN2 promoter and contained either mutated or unmutated EBE sites (#5-8) as indicated. Each probe was dsDNA and 5′ biotinylated. (**B**) Electro-mobility shift assays (EMSA) from nuclear lysates were generated from osteoblasts that were treated with TGF-β1 (5 ng/ml) for 2 hrs. Nuclear protein binding to the wild type and mutated EBE sites in the CTGF promoter was assessed using 5 µg of nuclear lysates. The lane number above each well corresponds to the probe used for that reaction. The experiment was repeated four times with similar results.

**Figure 6:**
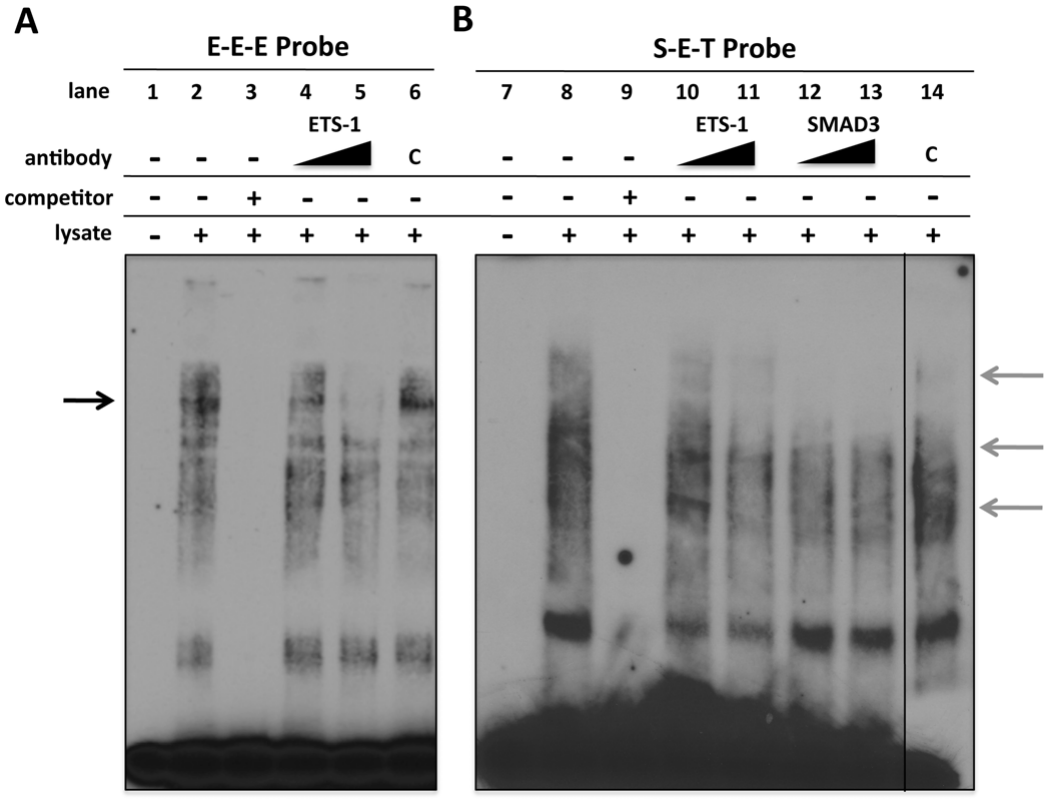
Ets-1 binds to EBE sites in the CCN2 promoter in osteoblasts. Electro-mobility shift assays (EMSA) from nuclear lysates were generated from osteoblasts that were treated with TGF-β1 (5 ng/ml) for 2 hrs. (**A**) Nuclear protein binding to the wild type E-E-E (lanes 1–6) (this probe contains EBE # 6-8; for probe design see Figure 6) probe was assessed using 5 µg of nuclear lysates for each reaction. In some reactions, Ets-1 antibody was added at increasing concentrations (1 ug of antibody in lane 4; Two micrograms of antibody in lane 5) to test for Ets-1/probe interaction. Control antibody (2 ug) was also used (lane 6; C). In some cases, probe only (lane 1) or a molar excess of unlabeled probe (lane 3) was also used to demonstrate specificity. (**B**) Nuclear protein binding to the wild type S-E-T (lanes 7–14) (this probe contains EBE#5; for probe design see Figure 6) probe was assessed using 5 µg of nuclear lysates for each reaction. In some reactions, Ets-1 antibody was added at increasing concentrations (1 ug of antibody in lane 10; 2 ug of antibody in lane 11) to test for Ets-1 protein/probe interaction or Smad 3 antibody (1 ug of antibody in lane 12; 2 ug of antibody in lane 13) to test for Smad 3 protein/probe interaction. Control antibody (2 ug) was also used (lane 14). In some cases, probe only (lane 7) or a molar excess of unlabeled probe (lane 9) was also used to demonstrate specificity.

Ets-1 regulates transcription of target genes in a tissue specific manner by directly binding DNA at highly conserved GGA (A/T) consensus sequences called Ets-1 binding motifs (EBE) [39], [40], [41]. Using bioinformatic analysis, we identified eight putative EBE sites in the CCN2 proximal promoter (Figure 3A). In order to determine the contributions of each of the EBE site, a site-directed mutagenesis approach similar to that of Nakerakanti et al was employed. [45]. Multiple promoter mutation constructs were created that contained point mutations in the eight putative EBE sites (Figure 3B) and cloned upstream into a pGL3 luciferase reporter construct. Using a luciferase-based reporter approach that we previously established in our laboratory [22], we found that mutation of EBE sites #4-8 significantly impaired CCN2 promoter responsiveness to TGF-β1 (Figure 4) with sites #5, 6, 7, and 8 having the greatest effect. In addition, we found that mutation of EBE sites in closer proximity to either the TGF-β response element (TRE) and/or the SBE had a more dramatic effect on CCN2 induction, suggesting potential synergetic interaction of these EBE sites with the TRE, SBE, or both. In order to determine if mutation of these sites prevented proteins from osteoblast nuclear lysates from binding these mutated EBE sites, electro-mobility shift assays (EMSA) were conducted using probes harboring the mutated or wild type versions of EBE sites #5-8 (Figure 5A). We found that nuclear proteins could bind the EBE sites in a TGF-β1 inducible manner (data not shown), and importantly that mutation of these EBE sites impaired normal protein binding (see Figure 5B: lane 1 vs. 2; 3 vs. 4; 4 vs. 5, 6 and 7). Interestingly, mutation of specific EBE sites, such as EBE #7, appeared to have a greater effect on protein binding affinity for adjacent EBE sites (see effects on EBE #6 and #8, Figure 5: lane 6), suggesting that this site mediates protein complex formation on adjacent EBE motifs. To validate that these sites were indeed bona fide EBE sites and to specifically determine which complexes were the result of Ets-1 protein binding, a super-shift EMSA approach was utilized. For these studies, EMSAs were conducted, as previously described, using the unmutated probes E-E-E (containing EBE #6-8) and S-E-T (containing EBE#5) (Figure 5A) however in these reactions we included Ets-1 specific antibody. As seen in Figure 6A, addition of the Ets-1 antibody to the E-E-E probe reactions inhibited complex formation (indicated by black arrow) in a dose-dependent manner (lane 4 [1 ul Ets-1 antibody] vs. lane 5 [2 ul Ets-1 antibody]) compared to the no antibody lane (lane 2) and the control antibody lane (lane 6), demonstrating that Ets-1 binds the E-E-E probe. Importantly, this same weight band also disappeared when we mutated the specific EBE sites (Figure 5B), demonstrating that mutation of EBE sites prevents Ets-1 binding at all three EBE motifs in this probe. We also performed a super-shift EMSA using the S-E-T probe to test if Ets-1 binds the EBE site found in this probe (Figure 6B). Addition of the Ets-1 antibody to the S-E-T probe reactions also inhibited complex formation (indicated by right grey arrows) in a dose-dependent manner (lane 10 [1 ul Ets-1 antibody] vs. lane 11 [2 ul Ets-1 antibody]) compared to the no-antibody lane (lane 8) and the control antibody lane (lane 14), demonstrating that Ets-1 binds the S-E-T probe. Additionally, since the S-E-T probe also contains a SBE, we also tested if addition of Smad3 antibody inhibited complex formation using this same approach and found that it blocked similar bands as the Ets-1 antibody (see lanes 12 and 13) when we used the S-E-T probe. Addition of Smad 3 antibody also blocked several other complexes, demonstrating that Ets-1 and Smad 3 synergize to form these protein complexes on EBE #5.

## Discussion

Numerous studies have demonstrated that Ets-1 plays a role in osteoblast differentiation and bone development [41], [46], [51]. Ets-1 is expressed in osteoblast and pre-osteoblast cells [41], and Ets-1 expression can be induced in the osteoblast cell line, MC3T3-E1, and primary fetal rat calvaria cells by retinoic acid (RA), which is known to exert profound effects on skeletal growth and development, bone turnover, and induce specific cellular responses in bone cells [41]. In addition, Ets-1 interacts with the osteoblast transcription factor CbfA1 to regulate expression of the osteoblast matrix gene osteopontin [46]. More recently, in a model for distraction osteogenesis, Ets-1 was found to activate expression of alkaline phosphatase, a marker for osteoblast differentiation [51]. However, while studies have demonstrated that Ets-1 is important for osteoblast differentiation, its mechanism of action in these cells remains largely undetermined. While some of effects of Ets-1 on osteoblast differentiation may in part be attributed to its interaction with Cbfa1, identification of other downstream target genes and elucidation of the precise role Ets-1 plays in osteoblast differentiation remain an important area of investigation.

In this study we investigated the contributions of Ets-1 for induction of CCN2 by TGF-β1 in primary, fetal rat calvarial osteoblasts. CCN2 is emerging as an important factor in the induction and control of osteogenesis. CCN2 is highly expressed in active osteoblasts during osteogenesis [1], during fracture healing [2], [3], and in osteoblasts during bone formation and regeneration [4]. Additionally, recombinant forms of CCN2 can elicit osteoinductive responses in bone, enhance osteoblast differentiation, and induce bone formation [2], [5]. However, the signaling mechanisms that control CCN2 expression, specifically in the context of osteoblasts, are poorly understood. We have previously identified two promoter motifs, the TGFβ response element (TRE/aka the BCE) and the SBE, that are both essential for CCN2 promoter activation by TGF-β1 in osteoblasts [22], [23]. In addition, we have demonstrated that CCN2 protein induction requires contributions of the Src, Smad, and Erk signaling pathways, and that all of these pathways converge at the level of the promoter, as Erk is necessary for both TRE and SBE transactivation [22], [23]. Several members of the Ets-1 family are downstream signaling targets of the Ras-MAPK pathway [37], and in certain cell types, Ets-1 is directly phosphorylated by Erk and functions to transactivate downstream Erk responsive genes [37], [38], [39], [40]. Given the requirement of Erk signaling in the context of CCN2 induction by TGF-β1 in osteoblasts, we investigated the potential role of Ets-1 in CCN2 induction by TGF-β1 in osteoblasts.

Ets-1 is known to be a downstream signaling effector for several TGF-β1 responsive genes [42], [43], [44], and more recently Ets-1 has been shown to function downstream of TGF-β1 for CTGF induction in fibroblasts [45]. In this study, we demonstrated that TGF-β1 treatment induces Ets-1 expression, and that this is concomitant with induction of CCN2 in osteoblasts (Figure 1A). Importantly, when we transiently over-expressed Myc-tagged Ets-1 protein using a mammalian expression vector, we were able to induce CCN2 expression (Figure 1B), and this was enhanced with simultaneous Ets-1 over-expression and TGF-β1 treatment. To confirm that Ets-1 was required for CCN2 induction by TGF-β1, we used Ets-1 siRNA to impair Ets-1 expression (Figure 1C) and found a significant reduction in CCN2 induction by TGF-β1 (Figure 1C), demonstrating the requirement of Ets-1 for CCN2 induction by TGF- β1 in osteoblasts.

In addition to CCN2 expression, we also assessed the requirement of Ets-1 for CCN2 promoter activation by TGF-β1 in osteoblasts. When we over-expressed Ets-1 protein we found that it induced CCN2 promoter activity at a level similar to TGF-β1 treatment, and that this promoter activity was significantly enhanced by simultaneous TGF-β1 treatment (Figure 2A), suggesting that Ets-1 and TGF-β1 synergistically activate the CCN2 promoter in osteoblasts. When we blocked Ets-1 with specific Ets-1 siRNA, we significantly impaired TGF-β1 induced CCN2 promoter activation (Figure 2B) further demonstrating that Ets-1 is required for promoter activation by TGF-β1 in osteoblasts.

The Ets-1 DNA binding domain (ETS) consists of 85 amino acids which recognize a highly conserved consensus sequence, GGA(A/T), called an Ets-1 binding motif (EBE) [39], [40], [41]. Using bioinformatic analysis, we identified eight putative EBE sites in the CCN2 proximal promoter in various degrees of proximity to the TRE and SBE (Figure 3). EBE sites #1-4 are novel and have never previously been described, while EBE sites # 5-8 have been identified by other groups [45], [48], but are novel in our system as their contribution to CCN2 induction by TGF-β1 in osteoblasts has not been described. In order to determine the relative contributions of these motifs to CCN2 induction by TGF-β1, we employed a site-directed mutagenesis approach (Figure 3B) and found that mutation of EBE sites #4-8 significantly impaired CCN2 promoter responsiveness to TGF-β1 (Figure 4), with sites #5-8 having the greatest effect. Interestingly, mutation of EBE sites in closer proximity to either the TRE or SBE had a more dramatic effect on CCN2 promoter activation by TGF-β1, suggesting potential synergetic interaction of these EBE sites with the TRE, SBE, or both. These results are consistent with a previous study in fibroblasts [45], where mutation of EBE sites #5 and #6 significantly impaired TGF-β1 induction of CCN2, however in that study, in contrast to what we found in osteoblasts, mutation of EBE sites #7 and #8 had no effect on CCN2 induction by TGF-β1, demonstrating that specific EBE sites differentially contribute to CCN2 induction by TGF-β1 induction in a cell type specific manner.

When we tested if mutation of these specific sites (#5-8) prevented protein binding, we found that nuclear proteins could bind each EBE site in a TGF-β1 inducible manner (data not shown), and importantly that mutation impaired protein binding (Figure 5). Interestingly, mutation of specific EBE sites, such as EBE #7, appeared to have a greater effect on protein binding affinity for adjacent EBE sites (see effects on EBE #6 and #8, Figure 5: lane 6), suggesting that this site mediates protein complex formation on adjacent EBE motifs. Previous studies in other cell systems have demonstrated a similar correlation between binding affinity and EBE spacing, where EBE motifs in closer proximity to one another have a higher affinity for Ets proteins then when motifs are spaced farther apart [52]. In addition, it is also known that the nucleotide sequences flanking these EBE motifs can influence the binding of particular Ets proteins [53], [54], and that Ets-1 protein can also bind to single or dual EBE motifs, with binding to multiple motifs considered more stable [55]. Our results clearly show that EBE #7 appears to be a critical site for protein complex formation, and that this protein complex formation is the result of Ets-1 binding (Figure 6A); however, how it coordinates/stabilizes protein complex formation and the identities of all the proteins involved in these complexes is an important area of investigation for future studies.

To determine if these putative motifs were bona fide Ets-1 binding sites and to specifically determine which complexes resulted from Ets-1 protein binding, a super-shift EMSA approach using Ets-1 antibody was employed. We tested Est-1 binding on EBE sites #6-8 found on the E-E-E probe (Figure 6A) and Ets-1 binding on EBE site #5 found on the S-E-T probe (Figure 6B). We found that we could block protein complex formation on all EBE sites using Ets-1 antibody, demonstrating that Ets-1 binds at these sites; moreover, we were also able to block the same weight band that disappeared when we mutated the specific EBE sites (Figure 5B), demonstrating that mutation of EBE sites prevents Ets-1 binding at all four EBE motifs in these probes. Transcriptional activation by Ets-1 protein can function in a combinatorial manner through association with other transcription co-factors to transactivate target genes [39], [40], [46], [47]. Recently, Ets-1 was found to synergize with Smad 3 to activate CTGF expression in fibroblasts [48], and we recently demonstrated the requirement of Smad 3 for CCN2 induction by TGF-β1 in osteoblasts [22]. In order to determine if Smad 3 played a role in Ets-1 biding in osteoblasts, we also conducted super-shift experiments using our S-E-T probe (EBE #5) and Smad 3 antibody. Interestingly, addition of Smad 3 antibody also blocked similar bands as the Ets-1 antibody (Figure 6B) in these experiments, suggesting that Ets-1 and Smad 3 synergize to form these protein complexes at EBE #5. Whether Smads and Ets-1 bind prior to CCN2 promoter binding or whether this interaction is secondary to complex formation on the promoter is unknown. Additionally, the role of Smads on Ets-1 binding of other EBE sites including EBE #6-8 is unknown, and studies addressing these interactions are warranted in the future.

In conclusion, this study demonstrates that Ets-1 is an essential downstream signaling component for CCN2 induction by TGF-β1 in osteoblasts, and that specific EBE sites in the CCN2 promoter are required for CCN2 promoter transactivation in osteoblasts. This is the first study that has explored the contributions of Ets-1 for TGF-β1 induction of CCN2 in osteoblasts. Identification of CCN2 as a downstream target of Ets-1 reveals a potential mechanism that may help unravel some of the effects of Ets-1 on osteoblast growth and differentiation. In addition, we have identified novel EBE sites in the CCN2 proximal promoter that are required for CCN2 induction by TGF-β1 in osteobalsts and have demonstrated that these EBE sites cooperate to achieve protein binding. We have also found that Ets-1 and Smad 3 synergize to achieve protein binding in EBE sites in close proximity to the SBE. Future studies will be necessary to assess the role that these EBE motifs play on SBE and TRE occupancy and the potential interaction of other nuclear co-factors like Smads, p300 and CBP on EBE transactivation in osteoblasts.

## Materials and Methods

### Ethics Statement

No human subjects were used in this study. This study was approved by the IACUC review board of the University of Scranton, Scranton PA. (IACUC approval #13-07). All animals were handled according to national and international guidelines following the principles in the NIH Guide for the Care and Use of Laboratory Animals (U.S. Department of Health and Human Services, Publ. No. 86-23, 1985) and in accordance with principles established in the Weatherall report.

### Reagents

Transforming Growth Factor-β1 (TGF-β1) was purchased from Calbiochem and reconstituted as 1 µg/ml in 4 mM HCl with 0.1% bovine serum albumin. Anti-actin antibody was purchased from Sigma. Anti-CTGF and anti-ETS-1 antibodies were purchased from Santa Cruz. Horseradish peroxidase conjugated anti-rabbit and anti-mouse IgG antibodies were obtained from Pierce. Myc-DDK-Tagged Ets-1 expression vector was purchased from OriGene.

### Source of Animals

Primary osteoblasts were derived from bone (calvaria) of neonatal Sprague Dawley rats purchased from Charles River. All animals were handled according to the principles in the NIH Guide for the Care and Use of Laboratory Animals (U.S. Department of Health and Human Services, Publ. No. 86-23, 1985) and guidelines established by the IACUC of the University of Scranton (Scranton, PA).

### Primary Osteoblast Cell Culture

Primary osteoblast cultures were obtained using neonatal rats as previously described [2], [5], [13]. Primary cells were isolated from parietal calvaria from which the periosteum and cranial sutures were removed to reduce non-osteoblast cell contamination. Calvaria pieces were subject to five sequential digestions of 5, 15, 15, 25, and 25 min at 37°C in a shaking water bath with 0.1% collagenase-P (Roche)/0.25% trypsin (Mediatec). The first two digestions are performed to remove non-osteoblast cells and are discarded. Osteoblast-enriched cell populations were obtained from the 3^rd^–5^th^ digestions of the calvarial pieces. These cells were plated in 100 mm dishes (Falcon) at 5×10^5^ cells/plate in osteogenic media consisting of Earle's Minimal Essential Medium (EMEM; Mediatec) supplemented with 10% fetal calf serum (FCS; Mediatec), 50 µg/ml ascorbic acid (Sigma) and 10 mM β-glycerophosphate (Sigma). The cells were incubated at 37°C with 5% CO_2_ with a change of media every three days until they reached 80% confluence. Cells were sub-cultured under identical conditions for utilization in experiments following the third passage. We have previously shown that these culture conditions result in an enhanced (>90%) population of cells committed to the osteoblast lineage using specific markers of osteoblast differentiation (e.g. Runx2, Osterix) [2], [13].

### Promoter Constructs

The previously characterized pGL3-W787 vector [22] was used to construct multiple mutation constructs using previously defined mutations [45] for each identified EBE site. Point mutations were introduced using QuickChange Site directed mutagenesis kit (Stratagene) the following primers (IDT): EBE -1: 5′ CATCCAAGAGACTACAGTCCCATGAATTAAAAAAAATCTAAAACAGTGAAAAAGA -3′, 5′- TCTTTTTCACTGTTTTAGATTTTTTTTAATTCATGGGACTGTAGTCTCTTGGATG -3′; EBE 0: 5′- GAACTGGTAAAGCGATTTTTAAGAAGTTAAGATTCGAGAAATAATCCTTGTTCA -3′, 5′- TGAACAAGGATTATTTCTCGAATCTTAACTTCTTAAAAATCGCTTTACCAGTTC -3′; EBE 1: 5′- TCATGTATTTCTAAGTTATATTTCATCATTAAGGGTGCGAAGAGGATACAG -3′, 5′- CTGTATCCTCTTCGCACCCTTAATGATGAAATATAACTTAGAAATACATGA -3′; EBE 2: 5′- GCTGGAAACACAACGCCTTTTTTTTTCTTAATGGCCAGCTAAAGTG -3′, 5′- CACTTTAGCTGGCCATTAAGAAAAAAAAAGGCGTTGTGTTTCCAGC -3′; EBE 3: 5′- GCCAGCTTTTTCAGACGGATTAATGTGGAGTGTCAAGGGG -3′, 5′- CCCCTTGACACTCCACATTAATCCGTCTGAAAAAGCTGGC -3′; EBE 4: 5′- GTGTGAGTTGATGAGGCATTAAGGTGGGGAGGAATGCG -3′, 5′- CGCATTCCTCCCCACCTTAATGCCTCATCAACTCACAC -3′; EBE 5: 5′- GGCAGGAAGGTGGGGATTAATGCGAGGAATGTCC -3′, 5′- GGACATTCCTCGCATTAATCCCCACCTTCCTGCC -3′; EBE 6: 5′- AAGGTGGGGAGGAATGCGATTAATGTCCCTGTTTGTGTAG -3′, 5′- CTACACAAACAGGGACATTAATCGCATTCCTCCCCACCTT -3′. Mutations were confirmed with sequencing in both directions (Genewiz).

### Protein Isolation and Western Blotting

2×10^6^ cells/100 mm culture dish were washed twice in PBS and harvested from culture dishes with RIPA buffer (Pierce) according to the manufacturer's instructions. The whole cell lysates were stored at −20°C for later Western blot studies. Nuclear lysates were obtained using the NE-PER Nuclear Protein Extraction Kit (Pierce) as per the manufacturer's instructions. Total protein concentrations were measured using the BCA Protein Assay Reagent Kit (Pierce) according to the manufacturer's instructions. Twenty µg of protein from each sample were mixed with 1× NuPAGE LDS Sample Buffer and 1× NuPAGE Sample Reducing Agent (Invitrogen) and heated at 95°C for 7 minutes. Samples were subjected to electrophoresis on NuPAGE 10% Bis-Tris gels (Invitrogen) and transferred to Hybond ECL membranes (GE Healthcare) by electroblotting. After 1 hour blocking in 5% BSA or 3% dry milk/0.5% BSA (per antibody instructions) at room temperature, blots were incubated with one of the following primary antibodies: Ets-1 (1∶200), actin (1∶5000), and CTGF (1∶200), and then with the corresponding HRP-conjugated secondary antibody (1∶20,000). Antigens were detected using the Pierce Supersignal West Pico Chemiluminescent Substrate System.

### Electro-Mobility Shift Assay

Nuclear extracts from TGF-β1-treated cells were prepared following the nuclear protein separation protocol described above. The electro-mobility shift assays and used in this study were performed as previously described [22]. Biotinylated oligonucletide probes were purchased from Integrated DNA Technologies. The binding reaction was composed of 5 µg of nuclear extract, 60 fmol of labeled probe, and binding buffer (20 mM Tris (pH8.0), 0.5 mM EDTA, 10% glycerol and 1 mM DTT). After incubating at RT for 30 min the entire sample was loaded on a NuPAGE 7% Tris-Acetate gel (Invitrogen), subjected to electrophoresis in native tris-glycine running buffer, and transferred to Biodyne B Pre-Cut Modified Nylon Membranes (Thermo) by electroblotting. Shifts of the biotinylated probes were detected using the Chemiluminescent Nucleic Acid Detection Module (Thermo).

### Luciferase Assays

Luciferase activity was determined using the Dual-Glo luciferase assay (Promega) according to the manufacturer's instructions. Primary osteoblasts were plated in a 96-well microplate (2.4×10^4^ cells/well), transfected with 0.4 µg of the unmutated pGL3-W787 reporter vector or one of the eight EBE mutant vectors, and co-transfected with 0.2 µg of a Renilla (internal control) luciferase vector. Following transfection, the cells were serum starved overnight and treated with TGF-β1 (5 ng/ml) for an additional 24 hours. Luciferase activity was measured using a SpectraMax M5 Microplate Reader. Relative luciferase activity was expressed as a ratio of firefly/renilla luminescence values. All samples were normalized to an untreated (cells only) or mock treated (empty vector or diluent only) control reaction.

### RT-PCR

RT-PCR was performed as previously described [56]. Briefly, RNA was extracted from primary osteoblasts that were transfected with 100 nM of Ets-1 siRNA or control siRNA (control) for 48 hrs. Following transfection with siRNA, the cells were serum starved for 24 hrs and then treated with 5 ng/ml of TGF-β1 for 24 hrs. Total RNA was harvested using Trizol (Invitrogen) according to the manufacturers directions. First strand synthesis was performed using SuperScript III (Invitrogen) according to the manufacturers directions and amplified using previously described primers and conditions for Ets-1 and GAPDH [56].

### Data Analysis

For all quantitative data, analysis of variance (ANOVA) was employed to evaluate the effect of one variable on two or more independent groups. In the event of a significant group effect, individual pairs of means were compared using the Bonferroni post-hoc test. Data were calculated as mean+SEM, and in some cases, converted to percent of control. A value of p<0.05 was used to determine whether differences were statistically significant.
